# Impact of clinical and psychological factors associated with depression in patients with rheumatoid arthritis: comparative study between Germany and Brazil

**DOI:** 10.1007/s10067-020-05470-0

**Published:** 2020-10-26

**Authors:** Harriet Morf, Geraldo da Rocha Castelar-Pinheiro, Ana Beatriz Vargas-Santos, Christoph Baerwald, Olga Seifert

**Affiliations:** 1 Department of Rheumatology, University Hospital Erlangen, Ulmenweg 18, 91054 Erlangen, Germany; 2 Department of Internal Medicine 3 and Institute for Clinical Immunology, University Hospital Erlangen, Maximilianspl. 2, 91054 Erlangen, Germany; 3 Department of Internal Medicine, Rheumatology Unit, Universidade do Estado do Rio de Janeiro, R. São Francisco Xavier, 524-Maracanã, Rio de Janeiro, Brazil; 4 Department of Rheumatology, University Hospital Leipzig, Liebigstraße 20, 04104 Leipzig, Germany

**Keywords:** Rheumatoid arthritis, Depressive symptoms, Psychoeducational strategies

## Abstract

**Objective:**

To investigate the prevalence of depressive symptoms and its association with clinical and psychological factors in patients with rheumatoid arthritis (RA) in Germany and in Brazil.

**Method:**

A convenience sample of 267 RA patients, 176 from Germany (age 62.4 ± 12.3 years) and 91 from Brazil (age 56.3 ± 12.6 years), was used in this cross-sectional study. The following questionnaires were used: Beck Depression Inventory (BDI), painDETECT test, Perceived Stress Questionnaire, fatigue questionnaire (FACIT), Health Assessment Questionnaire Disability Index (HAQ-DI), and the SF–36 questionnaires (Short-Form 36 Health Survey). Disease activity score (DAS 28-CRP) and visual analogue scale (VAS) for pain were also evaluated. Statistical analysis is based on comparison of means and proportions. Statistical significance for non-normal data was evaluated by non-parametrical tests.

**Results:**

Depressive symptoms were more prevalent in the Brazilian sample (44% vs 22.9%, *p* = 0.025). Compared to German patients, the Brazilian ones also experienced more pain (current pain status on VAS: 4.67 ± 3.4 vs 3.67 ± 2.31 respectively, *p* < 0.01), were physically more limited (1.89 ± 1.85 vs 1.01 ± 0.75, *p* = 0.012), and had higher C-reactive protein levels (7.78 ± 18.3 vs 5.82 ± 10.45, *p* = 0.028). Despite receiving a more intensive treatment, German patients presented similar disease activity when compared to Brazilian patients (DAS28-CRP: Brazil 3.4 ± 1.5 vs Germany 3.3 ± 1.3, *p* = 0.307).

**Conclusion:**

Depressive symptoms are frequent in RA patients from different countries and interact with psychological disorders and the experience of pain. They contribute negatively to their well-being suggesting the need for psychoeducational strategies.

**Table:** 

**Key Points** • *New psychoeducational strategies for RA management.*• *Higher inflammation marker in rheumatoid arthritis patients is associated with depression.*• *Medical treatment in RA influences depressive symptoms.*• *Depressive symptoms are dependent on population group.*• *High disease activity is related to depression.*

## Background

Rheumatoid arthritis (RA) is a chronic inflammatory disease of unknown aetiology. It leads to pain and swelling and may progress to destruction of peripheral joints. Although a variety of conventional synthetic, biological, and more recently developed, targeted synthetic disease modifying anti-rheumatic drugs (cs, b, tsDMARD) exist, the treatment of the disease is still a challenge, requiring close monitoring for a long follow-up period [1]. The female predominance in younger ages diminishes with rising age and vanishes in people aged 75 years and above [2]. The prevalence of RA differs worldwide, as do the incidence rates, ranging from the median annual incidence of 13.4 cases per 10,000 inhabitants in Brazil to 38 cases per 10,000 inhabitants (range 31 to 45) for North American countries. In North European countries, RA is known with an incidence of 29 cases per 100, 000 inhabitants (range 24 to 36) [3, 4].

RA is associated with psychological disorders, especially depression [5]. About 13–20% of RA patients worldwide have clinically relevant depression compared with 6% in the general population [6, 7]. It was shown that depression in RA patients has a high variability between countries ranging from 2% in Morocco up to 33% in the USA [8]. Mental disorders including depression are the most frequent comorbidities in RA [9]. In clinical practice, depressive symptoms and corresponding disorders are common in RA with a recent meta-analysis reporting an estimated 16.8% of RA patients as having a major depressive disorder [10]. The coexistence of immune-mediated inflammatory diseases with depression has been recognized for a long time [11]. However, until now, it is not clear if inflammation markers can also be used as markers for depression. Also unclear is the influence of medications on RA patients with depressive symptoms. Thus, detecting and addressing depression in RA patients are highly relevant and need to be part of optimal care of these patients [12]. Therefore, we studied the prevalence of depressive symptoms and their associations with parameters of disease activity, severity, and psychological factors in a cohort of RA patients from Germany and Brazil.

## Material and methods

### Study design and settings

This was a cross-sectional study carried out in two RA outpatient clinics: one from Leipzig, Germany, in 2011 and 2012, and one from Rio de Janeiro, Brazil, in 2013. Data were collected from medical records and from questionnaires applied during the scheduled visit for routine care.

### Patients

RA was classified according to the 2010 American College of Rheumatology (ACR)/European League against Rheumatism (EULAR) Classification Criteria for Rheumatoid Arthritis [13]. Patients of both sexes and age ≥ 18 years were included. Exclusion criterion was the presence of a chronic degenerative neurological disease. All patients provided written informed consent. The study was approved by the medical ethics committee of the University of Leipzig.

### Variables

#### Demographic and lifestyle variables

Demographic data were collected during the medical visit and included age, sex, marital status, history of having children, and employment status. Only German patients were asked about their smoking habits.

### RA disease activity and psychological evaluation

#### RA characterization

The current RA disease activity was determined by the disease activity score based on a 28 joint assessment and C-reactive protein level (DAS28-CRP), performed as routine care. Disease duration was verified during the medical visit. The characterization of erosive disease was based on the results of X-ray of hands and feet described in the medical records. The presence of rheumatoid factor or anti-citrullinated protein antibodies was also verified from medical records. ACPA was rarely measured in the Brazilian cohort because of the unavailability of the test. C-reactive protein (CRP) was collected when the required equipment was available during the medical visit. Ongoing RA treatment was assessed through levels of dosage of glucocorticoids, conventional synthetic disease modifying anti-rheumatic drugs (csDMARDs), and biological DMARD (bDMARDs).

The functional disability was assessed by the Health Assessment Questionnaire Disability Index (HAQ-DI) [14]. Pain intensity during the prior week (current pain) was evaluated using a 10 cm visual analogue scale (VAS) anchored by two verbal descriptors “no pain” (score of 0) to “worst possible pain” (score 10). Other parameters were worst pain ever (10 cm VAS) and the average of pain in the last 4 weeks (10 cm VAS).

#### Depression characterization

Depressive symptoms were evaluated by the Beck Depression Inventory (BDI) [15], with a score of less than 13 being considered as normal, 14–19 points being coded as mild or probable depression, 20–28 points being classified as moderate or definite depression, and more than 28 points being valued as severe depression [16]. Special training sessions on how to work with the Beck Depression Inventory were held by a psychiatrist prior to the study period at participating sites.

#### Additional questionnaires

Quality of life was evaluated by the Short-Form Health Survey (SF-36) [17]. Additionally, we utilized the painDETECT test [18] for the measurement of neuropathic pain; if the resulting score is higher than 18 points, the classifier for neuropathic pain is coded as ‘positive’ (probability > 90%). Stress was evaluated by the Perceived Stress Questionnaire (PSQ) [19]: more than seventy-five points is an index for stress [20]. Fatigue was estimated with the Functional Assessment of Chronic Illness Therapy–Fatigue (FACIT-Fatigue Scale) questionnaire [21]. All questionnaires were administered in the patients’ native languages and had been previously validated.

### Statistical methods

First, we compared the two populations concerning sociodemographic, clinical, incapacity, and treatment characteristics. We used means and standard deviations for the continuous variables and proportion for the nominal ones. The same tests were used for comparing depression symptoms, pains, stress, fatigue, and physical and mental health components of the SF-36. Kolmogorov-Smirnov test and graphics were used to inspect normal distribution of continuous variables. For non-normal distribution, we used the Mann-Whitney *U* Test instead of Student *t* test for statistical significance. In cases of more than two groups, Kruskal-Wallis test was used to compare the mean values. The statistical significance for nominal variables was tested by the Chi-square and the Fisher’s test. *p* values were two-sided and considered statistically significant if ≤ 0.05. Spearman correlation was estimated for the correlation between psychological markers and depressive symptoms.

The analyses were performed using IBM SPSS Statistics 20 Windows (SPSS Inc., Chicago, Illinois, USA) and Excel Windows (Microsoft® GmbH, Unterschleißheim).

## Results

### Participants

The demographic characteristics of RA patients are presented in Table 1. Participants in the Brazilian sample were significantly younger than members of the German group and had more female and single patients. Distribution of employment status was similar between the samples.

**Table 1:** Characteristics of German und Brazilian RA patients (mean ± SEM or *n* (%))

	German patients *n* = 176	Brazilian patients *n* = 91	*p*
Age, years	62.43 ± 12.33	56.3 ± 12.62	< 0.001
Age > 65 years	87 (49.4%)	24 (26.3%)	< 0.001
Women	138 (78.4%)	84 (92.3%)	< 0.001
Smoking	16 (12.9%)	No information	
Marital status			
Married	114 (64.8%)	32 (35.2%)	0.001
Single	19 (10.8%)	32 (35.2%)	0.001
Divorced	9 (5.1%)	13 (14.3%)	0.001
Widow	26 (14.8%)	13 (14.3%)	0.001
Having children	140 (79.5%)	66 (74.2%)	0.013
Laboral situation			
Employed	56 (31.8%)	32 (35.2%)	1.00
Retired	116 (65.9%)	59 (64.8%)	0.283
Incapacitated	4 (2.2%)	0	
RA duration, years	14.35 ± 10.41	15.9 6 ± 10.28	0.234
Positive rheumatoid factor	104/148 (70.3%)	18/31 (60.0%)	0.042
Positive ACPA	91/125 (72.8%)	5/29 (17.2%)	0.000
Erosive disease	70 (51.1%)	40 (43.9%)	0.011
DAS28-CRP	3.30 ± 1.35	3.42 ± 1.51	0.307
CRP, mg/L	5.82 ± 10.45	7.78 ± 18.3	0.028
HAQ-DI	1.01 ± 0.75	1.89 ± 1.85	0.012
HAQ-DI > 0.5	65.5%	64.1%	0.908
Treatment			
Glucocorticoid (Gc), low dose	10 (5.7%)	2 (3.4%)	< 0.001
csDMARD ± Gc	68 (48.2%)	44 (74.6%)	0.003
bDMARD + csDMARD + Gc	33 (23.4%)	5 (8.5%)	1.00
bDMARD + csDMARD	5 (3.5%)	4 (6.8%)	1.00
bDMARD ± Gc	19 (13.5%)	3 (5.6%)	1.00

*bDMARD* biological disease modifying anti-rheumatic drug; *CRP* C-reactive protein; *csDMARD* conventional synthetic DMARD; *DAS* disease activity score; *HAQ-DI* Health Assessment Questionnaire Disability Index; *SEM* standard error of the mean

Most critically, there is no statistically significant difference between German and Brazilian RA patients with respect to mean disease duration, disease activity (DAS28-CRP), and percentage of patients with erosive disease (Table 1). In contrast, the mean of CRP level was 1.76 mg/L higher in Brazilian patients (*p* = 0.028) who had also a 0.88-point higher scores of disability score (HAQ-DI) compared to German patients (*p* = 0.012).

Concerning anti-rheumatic therapy, more German patients received low doses of glucocorticoid (5.5% vs 3.4%), while csDMARD (with or without glucocorticoid) were more prescribed to Brazilian patients (74.6% vs 48.2%). More patients in Germany (23.4%, compared to Brazil’s 8.5%) receive a combination therapy (biologics, csDMARD, and low dose prednisolone), but this difference is not statistically significant (*p* > 0.05) (Table 1).

### Main results

**Fig. 1:**
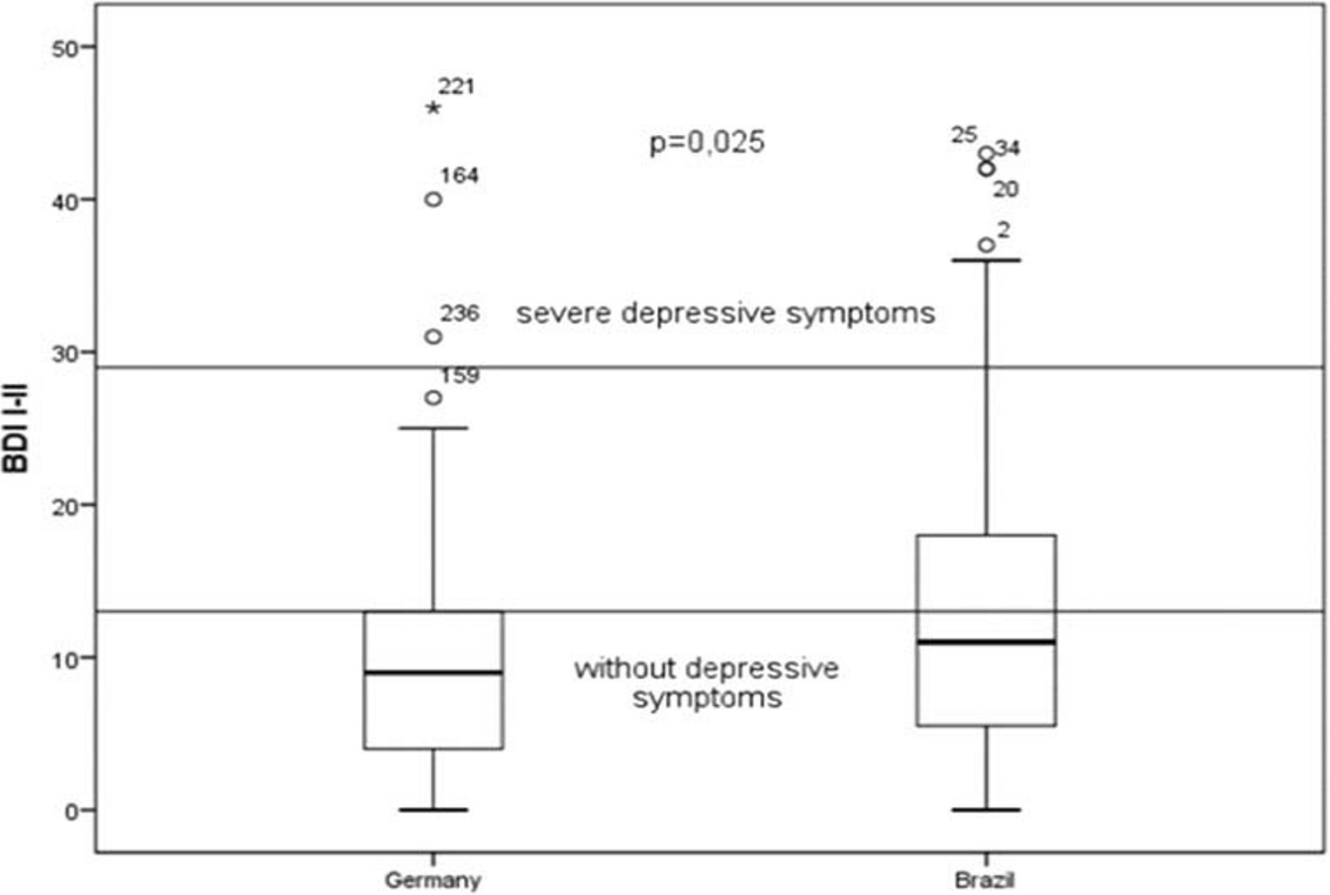
Difference in depression score between German and Brazilian patients; BDI, Beck Depression Inventory

BDI average scores are higher among Brazilian patients (13.0 versus 9.82; = 0.025 (Table 2 and Fig. 1). The percentage of RA patients with depressive symptoms (BDI score ≥ 13 points) in Brazil is almost twice as high as in Germany (44% vs 24.3%; *p* = 0.001) (Table 2). Although there were differences in the severity of depressive symptoms between the two groups, these differences did not reach statistical significance, probably due to sample size.

**Table 2:** Psychological characteristics of German and Brazilian RA patients

Parameters	German Patients *n* = 176	Brazilian Patients *n* = 91	*p*
BDI, mean ± SEM*	9.82 ± 7.3	13.0 ± 10.2	0.025**
BDI score, *n* (%)*			
≤ 13 (normal)	131 (75.7%)	51 (56.0%)	0.001
14–19 (mild or probable depression)	25 (14.5%)	21 (23.1%)	0.40***
20–28 (moderate depression)	14 (8.1%)	12 (13.2%)	
≥ 29 (severe depression)	3 (1.7%)	7 (7.7%)	
painDETECT, mean ± SEM	18.3 ± 6.29	20.54 ± 10.0	0.002**
Neuropathic pain (painDETECT score > 18), *n* (%)	85 (48.3)	53 (57.6)	0.001
VAS for current pain	3.67 ± 2.3	4.67 ± 3.4	0.016
VAS for worst pain	5.42 ± 2.67	6.56 ± 3.5	< 0.001
VAS for pain in the last 4 weeks	4.04 ± 2.04	4.59 ± 3.18	0.067
Perceived Stress Questionnaire (PSQ)	55.2 ± 16.5	37.10 ± 6.73	< 0.001**
Fatigue (FACIT-F)	37.8 ± 10.8	37.2 ± 10.0	0.484
Physical health component summary-SF-36 (PCS)	32.4 ± 19.43	33.7 ± 11.9	0.559
Mental health component summary-SF-36 (MCS)	51.4 ± 11.4	46.5 ± 12.2	0.002**

*BDI* Beck Depression Inventory; *SF-36* Short-Form Health Survey; *VAS* visual analogue scale

*Three missing patients in German sample; **statistical significance was calculated using the Mann-Whitney *U* test; ***p value for comparing the three levels of severity between countries

The frequency of depressive symptoms measured on the BDI score was twice as high in Brazilian women (45%) compared to German women (24%) (*p* = 0.02).

Brazilian patients scored significantly higher on the pain scales than German patients (Table 2). The VAS score was 1 point higher (*p* = 0.016) for current pain and 1.14 point higher for worst pain (*p* < 0.001). There was also a difference of 2.24 points for the painDETECT, being higher in the Brazilian sample (*p* = 0.002), leading to a prevalence of neuropathic pain (painDETECT score > 18 points) of 57.6% of Brazilian patients against 48.3% in German patients (*p* = 0.001).

There is no difference between the two groups concerning the prevalence of fatigue symptoms (Table 2). However, Brazilian patients had lower scores for the psychological quality of life (SF-36—mental health component summary (MCS)) compared to German patients (46.6 vs. 51.4; *p* = 0.002).

German patients who took glucocorticoids had significantly (*p* = 0.030) more depressive symptoms (83.8%) than the group without depressive symptoms (67.3%).

**Fig. 2:**
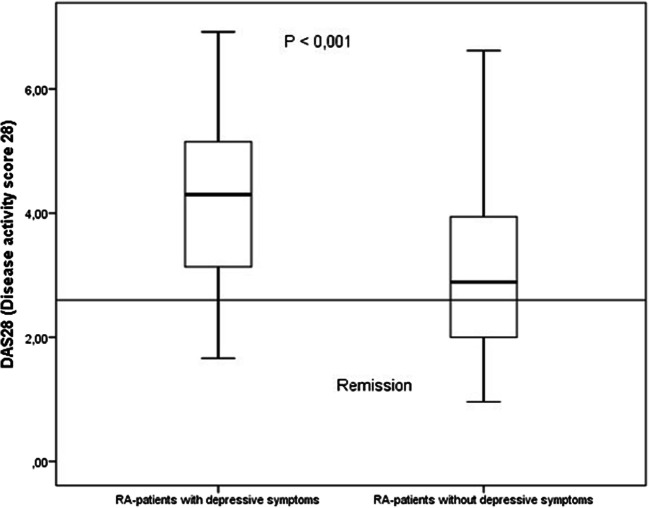
DAS28-CRP in German RA patients with and without depression

There was a significant correlation (*p* < 0.001) between depressive symptoms and disease activity in both groups of RA patients (Fig. 2). Interestingly, 12.6% of German patients with depressive symptoms were in clinical remission (DAS28-CRP < 2.3). While 26.8% of German patients with minimal to moderate depressive symptoms had active disease, only 5% without depressive symptoms had disease activity.

Table 3 depicts the correlation coefficients of BDI with different variables, stratified by country. These correlations were positive for pain variables, HAQ-DI, disease activity, and stress. In the case of SF-36, the correlation was inverse for both dimensions (physical and psychological). In the case of CRP, there was a small positive correlation for German patients, but no correlation (*p* = 0.553) for the Brazilian ones.

**Table 3:** Correlations between BDI and other variables

	German patients		Brazilian patients	
	Correlation	*p* value	Correlation	*p* value
Pain-DETECT	0.392	< 0.001	0.520	< 0.001
Current VAS	0.473	< 0.001	0.599	< 0.001
Worst VAS	0.471	< 0.001	0.598	< 0.001
4 Weeks VAS	0.487	< 0.001	0.498	< 0.001
HAQ-DI	0.446	< 0.001	0.552	< 0.001
DAS28-CRP	0.404	< 0.001	0.302	0.041
PSQ	0.656	< 0.001	0.621	< 0.001
MCS–SF-36	− 0.583	< 0.001	− 0.759	< 0.001
PCS–SF-36	− 0.651	< 0.001	− 0.330	0.012
CRP	0.226	0.003	− 0.107	0.553

*MCS–SF-36* mental health component summary of the Short-Form Health Survey

*PCS–SF-36* physical health component summary of the Short-Form Health Survey

## Discussion

In RA patients, the prevalence of depressive symptoms is rather high with varying results among different studies. Depending on the criteria used for depression, the prevalence was calculated between 16.8 and 38, 8%. Furthermore, depression was associated with poorer RA outcome [22].

Our study showed a markedly high prevalence of depressive symptoms in RA patients, particularly in the Brazilian cohort (every second patient), indicating a need for screening during routine clinical practice and corresponding follow-up procedures in case of depressive symptoms. Most of our patients showed mild depressive symptoms, while 17.5% of all patients had depressive symptoms so severe that they require special healthcare support in a psychiatric clinic.

Pain symptoms play an important role in the well-being of patients with rheumatic diseases [23]. Our study has demonstrated the association between depressive symptoms and chronic pain in Brazilian as well as in German RA patients [24]. This is consistent with other studies on chronic pain patients, e.g. in Brazil, where depression was observed in 35.2% of patients with chronic pain and was associated with lower quality of life in physical, mental, emotional, and social domains [25].

In our cohorts, RA disease activity was associated with depressive symptoms in both RA populations. Inflammation and trophic factors (brain-derived neurotrophic factor [BDNF], vascular endothelial growth factor, glial cell line-derived neurotrophic factor, and insulin-like growth factor-1) are associated with depression in the general population. One study showed that RA disease activity (DAS 28-CRP) and severity of fatigue were associated with the presence and intensity of depressive symptoms. [26].

In German RA patients, depressive symptoms are more prevalent among prednisolone users (83.8% vs. 67.3%). A previous study reported that RA patients with pain receive more medications despite a low disease activity, which was due to depression [27]. In addition to this stands the fact that glucocorticoids can lead to depression. Here, the hypothalamic-pituitary-adrenal axis plays a major role since 80% of patients with depressive symptoms suffer from hyperactivity of the HPA axis with a higher production of glucocorticoids [28, 29].

On the other hand, depression has a significant influence upon achieving remission in RA patients. Data from the British Society for Rheumatology Biologics Register showed that the presence of depression symptoms at biologic treatment initiation was associated with 20–40% reduced odds of achieving a good treatment response within 1 year. Experiencing symptoms of depression at the start of biologic treatment may reduce the odds of achieving a good treatment response and reduce the probability of disease control over time. Patients with a history of depression or reporting symptoms of depression according to the EuroQol five-dimension scale showed reduced improvement in tender and swollen joints, patient global assessment, and erythrocyte sedimentation rate (ESR). Therefore, depression should be managed as part of routine clinical care to optimize treatment outcomes in RA [30].

In turn, however, adequate anti-rheumatic therapy might help to tackle depressive symptoms in RA patients [31]. In this respect, the difference in the therapeutic setting between Brazilian and German RA patients might partly explain the differences observed in the percentage of RA patients with depressive symptoms. Of interest, the prevalence for depression in the general population is higher in Brazil than in Germany. Ours well as other studies could demonstrate that in Brazil the percentage of patients receiving a biologic therapy is lower compared to the German cohort [32]. It is true that it represents a considerable economic burden to increase the number of RA patients receiving therapy with biologics in Latin America. However, it has been calculated that the expanded use of biologic agents will result in cumulative cost net savings due to reduced indirect costs of RA [33]. Still, the compliance rate of an anti-TNF therapy is rather low in Brazil, i.e. after 1 year, 48.2% of RA patients continued using anti-TNF (± csDMARD) therapy, and at the end of the second year, only 23.1% of anti-TNF (± csDMARD) users were still on the medication [34]. There exists a difference in the healthcare systems, which might contribute to the observed differences. Due to different levels of access to public healthcare systems and greater economic inequality in Brazil, access to expensive drugs is limited. In Germany, the time between onset of symptoms and presentation to a rheumatologist could be reduced to 9 months [35, 36]. In contrast, recent studies in Brazil demonstrated a huge variation of the interval between onset of symptoms and diagnosis of a rheumatic disease, ranging in the REAL study from one to 457 months (median 12 months) and a mean of 28 months in a study from the country’s south [32, 37]. Both studies show that almost 80% of the RA patients in Brazil were of middle-low or low socioeconomic status, and the delay in diagnosing RA was associated with lower socioeconomic status and lower education level of the patients [38]. The sociodemographic comparison between our patient populations revealed differences for age and marital status of the patients, both potential influences for depressive symptoms in RA patients. In a population of older Brazilian adults, lower emotional support and depressive symptoms have been independently predictive for subsequent disability over a long term [39]. In addition, survival of married patients was longer due to a better life balance and a healthier lifestyle compared to single patients. In RA patients, social and emotional support is an important factor to reduce pain and depression [40].

### Limitations

Our study is an observational study with a few limitations. It was not possible to match the cohorts for socioeconomic factors. We used statistical tests and variables that were not adjusted for age, sex, or demographic status due to the relatively low number of RA patients. Furthermore, we could not evaluate the role of fibromyalgia as a differential diagnosis in our study. However, it might be difficult to differentiate between chronic pain, depressive symptoms, and fibromyalgia which have to be investigated in upcoming studies.

## Conclusion

Our data corroborate the idea that there are significant differences of psychological factors and prevalence of depressive symptoms between various countries. Our study indicates that RA-related depressive symptoms contribute to diminished psychological well-being in RA patients and points to the need for psychoeducational strategies that specifically target depression as part of an overall RA management program [41]. In this respect, our significant results should be confirmed by further studies.

## Data Availability

The authors confirm that the data supporting the findings of this study are available within the article or its supplementary materials
